# The Vaccination Bill

**Published:** 1898-03-26

**Authors:** 


### The Vaccination Bill.

The fact that the Government has introduced a Bill to amend the law with respect to vaccination raises the hope that some satisfactory settlement of this troublesome question will shortly be arrived at, and that our country will at last be provided with a degree of protection against small-pox comparable with that which other nations enjoy.

Notwithstanding all the labour and all the years spent on the subject by the Vaccination Commission, the discovery which has made it possible to frame the present Bill is one of the simplest things in the world, namely, that the addition of glycerine to vaccine lymph not only preserves it, so that the lymph so treated retains its full potency for a long time, but also greatly improves its quality, killing off those infectious organisms by which it is apt occasionally to be contaminated. This simple little discovery has changed the whole aspect of the question, for by its aid it has become possible to take vaccination to the people instead of compelling the people to come to the vaccination.

With ordinary untreated lymph the results of vaccination by means of "tubes" and "points" have been so inferior to those obtained by arm to arm or calf to arm vaccination that, notwithstanding the recognised evils connected with the plan of bringing the children together, "station vaccination" has always been maintained as the official system, and there can be very little doubt that dissatisfaction with this system has been one of the most important influences in furthering the anti-vaccinist crusade.

The broad line of distinction which the public vaccination system as worked at present draws between rich and poor, has a good deal to do, not, perhaps, with the agitation against vaccination—that is an affair of the agitator—but with the acceptability of the agitation among the masses. If Mr. Chaplin's Bill be passed, this cause of offence will be done away with, for the operation will be performed in the homes of the poor just as it now is in the homes of the rich.

The great point gained, however, by the use of glycerinated lymph is that by its means it will be possible to provide calf lymph for all. So long as vaccination with calf lymph could only be satisfactorily performed directly from the calf itself, its applicability was limited, but now that it is generally recognised that the lymph can be purified, preserved, packed up, and distributed to the four corners of the land, the one serious and reasonable objection to vaccination raised by thinking men is done away with. Mr. Chaplin has not been slow to take advantage of the opportunity thus afforded him, and it seems to us that his Bill is a distinct improvement on the recommendations of the Royal Commission. Instead of abolishing compulsion he maintains it, but he makes the *proviso* that the only form of vaccination which shall be compulsory shall be vaccination with calf lymph.

A matter of very great importance has yet to be considered, namely, who is to do the vaccination? There is much to be said in favour of the public vaccinator, and there can be little doubt that, so far as the past is concerned, private vaccination has often been a mere farce. But the conditions must be remembered. Public vaccination has been done under inspection and under the stimulation of rewards for good work, while private vaccination has never been inspected, and so far as reward is concerned the public, the great employer, has paid for what it wa ted, namely, just as much "vaccination" as would enable the practitioner to sign the certificate. Private vaccination has, in fact, been used to a large extent, not as a protection from small-pox, but as a protection from prosecution, and, under such circumstances, it is unfair to compare it with that done by public vaccinators. When the system of home vaccination gets to work, it is clear that some system of home inspection will have to be put in operation, and when this is done it is also clear that it will be as easy to inspect the performances of the private practitioner as those of the public vaccinator; and, with inspection, the one ought to be as good as the other. The advantage of utilising the services of the great army of private practitioners is one which can hardly be over estimated. Not only would their influence be enlisted in favour of efficient vaccination, but the stigma involved in the visit of the public vaccinator would be removed. We would even suggest a still further reason. The anti-vaccinists are dab hands at agitation, and it would be by no means outside and beyond their capacity to organise a system of espionage and dogging of the public vaccinator which should make his life a burden, and render the performance of the operation done to the tune of hooting crowds a terror and a scandal. Let vaccination, then, cease to be a public rite performed by public officials, and make it a part of ordinary private treatment, a matter between doctor and patient; let the official part of the business be restricted to providing the doctor with first-class vaccine, and paying him for first-class work, and then prosecuting those who refuse the benefits so freely offered to them. If this were done the chances are that in a few years time small-pox epidemics and small-pox hospitals would be things of the past.

